# A Treatise on Diseases of the Bones

**Published:** 1850-01

**Authors:** 


### ARTICLE V.

*A Treatise on Diseases of the Bones.*—By EDWARD STANLEY, F.R.S.; President of the Royal College of Surgeons of England, and Surgeon to St. Bartholomew's Hospital. Philad. Lea & Blanchard. 1849. pp. 286. 8 vo. (For sale by Jos. Keen & Bro., Chicago.)

This book came to hand as we were about to close the Bibliographical department of this number of the Journal, and we have had time to make only a very cursory examination of its pages. With the exception of the work of Dr. Gross, it is the first treatise which we recollect to have seen devoted entirely to a consideration of Diseases of the Bones.

Judging from the distinguished position of the Author, and his ample facilities for observation in this class of diseases, we should expect to find this volume one of uncommon interest and merit. In the next number we shall endeavor to furnish our readers a more extended notice. M.

